# Feedback control of arm movements using Neuro-Muscular Electrical Stimulation (NMES) combined with a lockable, passive exoskeleton for gravity compensation

**DOI:** 10.3389/fnins.2014.00262

**Published:** 2014-09-02

**Authors:** Christian Klauer, Thomas Schauer, Werner Reichenfelser, Jakob Karner, Sven Zwicker, Marta Gandolla, Emilia Ambrosini, Simona Ferrante, Marco Hack, Andreas Jedlitschka, Alexander Duschau-Wicke, Margit Gföhler, Alessandra Pedrocchi

**Affiliations:** ^1^Control Systems Group, Technische Universität BerlinBerlin, Germany; ^2^Research Group for Machine Design and Rehabilitation, Vienna University of TechnologyVienna, Austria; ^3^Hocoma AGVolketswil, Switzerland; ^4^NeuroEngineering and Medical Robotics Laboratory, NearLab, Department of Electronics, Information, and Bioengineering, Politecnico di MilanoMilan, Italy; ^5^Fraunhofer Institute for Experimental Software EngineeringKaiserslautern, Germany

**Keywords:** neuro-muscular electrical stimulation, neuroprosthetics, exoskeleton, feedback control, assistive technology, eye tracking

## Abstract

Within the European project MUNDUS, an assistive framework was developed for the support of arm and hand functions during daily life activities in severely impaired people. This contribution aims at designing a feedback control system for Neuro-Muscular Electrical Stimulation (NMES) to enable reaching functions in people with no residual voluntary control of the arm and shoulder due to high level spinal cord injury. NMES is applied to the deltoids and the biceps muscles and integrated with a three degrees of freedom (DoFs) passive exoskeleton, which partially compensates gravitational forces and allows to lock each DOF. The user is able to choose the target hand position and to trigger actions using an eyetracker system. The target position is selected by using the eyetracker and determined by a marker-based tracking system using Microsoft Kinect. A central controller, i.e., a finite state machine, issues a sequence of basic movement commands to the real-time arm controller. The NMES control algorithm sequentially controls each joint angle while locking the other DoFs. Daily activities, such as drinking, brushing hair, pushing an alarm button, etc., can be supported by the system. The robust and easily tunable control approach was evaluated with five healthy subjects during a drinking task. Subjects were asked to remain passive and to allow NMES to induce the movements. In all of them, the controller was able to perform the task, and a mean hand positioning error of less than five centimeters was achieved. The average total time duration for moving the hand from a rest position to a drinking cup, for moving the cup to the mouth and back, and for finally returning the arm to the rest position was 71 s.

## 1. Introduction

The consequences of Spinal Cord Injury (SCI) can be severe. Depending on the level of the lesion, SCI causes a loss of motor and sensory functions, and results in the immobilization of the patient. The level of lesion in SCI refers to the vertebrae in the spinal column affected by the injury. The higher the injury on the spinal cord, the more dysfunction can occur. Cervical (neck) injuries usually result in a full or partial tetraplegia (paralysis of the arms, legs, and trunk of the body below the level of the associated injury to the spinal cord). Individuals with a complete lesion at the C7 level or above (C6, C5, …) usually depend on attendant care for all daily life activities.

In SCI patients, the neural pathway from the Central Nervous System (CNS) to the muscles is interrupted. The injury may cause a complete or partial lesions of the upper and/or lower motor neurons. The upper motor neuron originates in the motor region of the cerebral cortex or the brain stem and carries motor information down to the lower motor neurons. All lower motor neurons (LMNs) related to voluntary movements are located in the ventral horn of the spinal cord and anterior nerve roots (spinal lower motor neurons) and innervate skeletal muscle fibers. They act as a link between upper motor neurons and muscles. In case of upper motor neuron lesions, Neuro-Muscular Electrical Stimulation (NMES) can be applied to the lower motor neurons that are still intact to cause artificial contractions of the innervated muscles (Sheffler and Chae, 2007). This will replace the lacking control signals from the CNS to the muscles.

Restoration of grasp function by NMES in spinal cord injured individuals has been realized by different research groups and is even available in form of commercial systems (for an overview see Popovic et al., 2002; Rupp and Gerner, 2007). Available neuroprostheses for grasping are able to restore the two most frequently used grasping styles: the palmar and the lateral grasp (Popovic et al., 2002). C7-C5 complete SCI subjects benefit the most from a grasping neuroprosthesis and achieve a high level of independence in Activities of Daily Living (ADL). These individuals have sufficient residual function of the proximal upper limb muscles that allow them to perform reaching tasks.

Injuries at the high C3 and C4 level result in a significant loss of function at elbow and shoulder level. Deltoid and the biceps muscles are innervated from the C5 and C6 level of the spinal cord. These muscles may be also denervated (lower motor neuron lesion), especially in case of C4 tetraplegia. However, the extent of denervation is likely to vary across individuals. The feasibility to restore shoulder and elbow functions at least partially by NMES was demonstrated by (Acosta et al., 2001) in persons with C3/C4 tetraplegia using percutaneous stimulating electrodes and by (Bryden et al., 2000) in persons with C5/C6 tetraplegia using a fully implanted stimulation system. However, the generated force in individuals with C3 and C4 SCI was not sufficient to hold the arm against gravity. In this context, it should also be noted, that a long lasting electrical stimulation of shoulder and arm muscles is overall not appropriate due to the fast fatigue of electrically stimulated muscles.

In order to enable reaching functions in individuals with SCI at C3 and C4 level, NMES-hybrid orthoses have been investigated. In Hoshimiya et al. (1989), a balanced forearm orthosis (BFO) was used for supporting arm motions. (Smith et al., 1996) used a suspended sling to provide shoulder joint stability, and Nathan and Ohry, (1990) applied mechanical splinting. All studies reported limited performance because of insufficient shoulder control. The stimulation was commanded by voice control (Nathan and Ohry, 1990), by breathing patterns (Hoshimiya et al., 1989) or by contralateral shoulder motion sensed by a position transducer (Smith et al., 1996).

Schill et al., (2011) developed the system OrthoJacket—an active NMES hybrid orthosis for the paralyzed upper extremity. The system combined NMES controlled grasping with an electrical/pneumatic actuation of shoulder movements and a flexible fluid actuator for support of elbow-joint movements. For control of the orthosis, EMG signals from arm muscles were acquired. This means that only individuals with some residual arm/hand functions could use this system. Furthermore, NMES was not used for movement generation at the shoulder or elbow-joint.

Within the EU project TOBI, a further NMES hybrid orthosis was developed to support both grasping and elbow-joint movements by NMES (Rohm et al., 2010). However, this system required sufficient residual shoulder function to be provided by the user. To avoid an excessive stimulation of the biceps muscle during holding tasks, the orthosis' elbow-joint was self-locking in direction of flexion and electrically de-lockable. A Brain Computer Interface (BCI) and a shoulder joystick at the non-supported side were provided as interfaces for the control of the orthosis.

In all existing systems, either NMES was applied in an open-loop manner using pre-defined stimulation patterns or the patient had to adjust the stimulation intensity by himself, e.g., via a position transducer at the contralateral shoulder or through EMG signals of preserved muscles. None of the systems allows the automatic positioning of the hand at arbitrary positions in the reachable workspace. In addition, deviations from the desired behavior, e.g., due to muscular fatigue, are not automatically compensated.

This study aims at developing a fully feedback-controlled arm neuroprosthesis for individuals with no or very weak residual arm and shoulder functions (such as persons with C3/C4 tetraplegia). In contrast to existing arm neuroprostheses, the proposed solution allows to position the hand at arbitrary desired positions within the reachable workspace. This arm neuroprothesis is a component of the modular assistive framework MUNDUS (Pedrocchi et al., 2013), that has been developed to support and recover arm and hand functions in severely impaired people. The arm reaching functionality can be extended by a robotic or NMES-based module for grasping assistance.

To reduce the amount of required stimulation for the arm and shoulder muscles, a passive light-weight exoskeleton supports the user in addition to NMES. The main purpose of the exoskeleton is the gravity compensation by a passive spring mechanism. In addition to this, the exoskeleton enables all joints to be locked for holding the arm at given positions without NMES. Thus, only point-to-point movements under gravity compensation have to be realized by means of artificial muscle activation, assuming no or insufficient residual motor control by the user over his/her arm and shoulder musculature.

Automatic control of NMES to achieve functional shoulder/arm movements is challenging due to the highly non-linear and time-varying behavior of the electrically stimulated muscles (Lynch and Popovic, 2008). Mimicking physiological movements would require to identify the musculo-skeletal system of the arm for each individual and each time the system is applied. This would require a long lasting calibration procedure infeasible in clinical environments or at home. For the use of NMES in stroke rehabilitation, Iterative Learning Control (ILC) has been proposed in order to generate precise functional reaching movements (Freeman et al., 2012). ILC demands a cyclic movement generation. After every movement cycle, an error trajectory with respect to a given reference movement will be determined and used to either update an open-loop applied stimulation pattern or to update the reference trajectory of an underlying feedback controller. The latter approach guaranties a sufficiently small tracking error even for initial ILC trials but again requires a detailed model in order to design the feedback controller. To avoid any huge calibration effort, we present a simpler movement generation strategy that involves sequential NMES control of all Degrees of Freedoms (DoFs) available in the exoskeleton.

The manuscript is structured as follows: in Section 2.1, an overview of the overall control system architecture is given. Sections 2.2 and 2.3 then describe the employed exoskeleton and the muscle actuation by NMES, respectively, in detail. In Section 2.4, we introduce the kinematic model of the exoskeleton and its parameter identification as well as required coordinate transformations used by the arm controller. In Section 2.5, the feedback controlled generation of arm movements is presented in detail. Then, in Section 2.6, we describe the experimental trials performed on healthy subjects to evaluate the performance of the control system. Section 3 summarizes the results in terms of the positioning error and execution times achieved in the validation trials. The article closes with a discussion and some conclusions.

## 2. Materials and methods

### 2.1. Control system architecture

**Figure 1:**
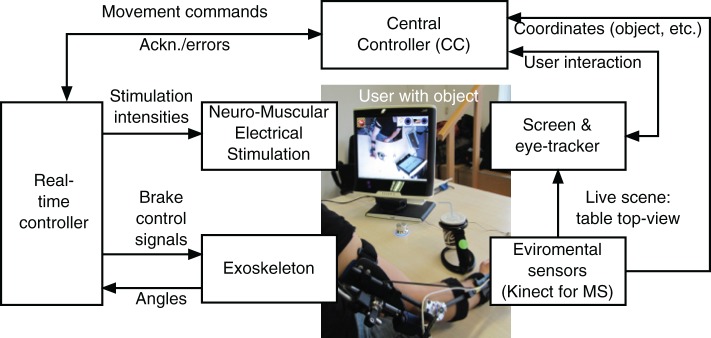
**System architecture for support of reaching function**.

The entire system developed for the support of the reaching movements is depicted in Figure 1. Potential users have no or very weak residual voluntary activation of arm, shoulder and hand muscles, but they can still control the head and gaze fixation. They usually sit in a wheelchair in front of a table. The target motions supported by the system are daily life activities, such as drinking, eating, brushing, touching the own body, pushing an alarm button, and moving an object on the table.

The arm/shoulder movements are induced by NMES while an exoskeleton guides the movement and supports the arm during static postures in absence of NMES. The control signals (stimulation intensities and on/off state of the exoskeleton brakes) are generated by a real-time controller that receives commands from the Central Controller (CC) implemented in form of a finite state machine. The central controller instructs the real-time controller to move the hand to a given target position in the reachable workspace. Sensors integrated in the exoskeleton measure joint angles that are used as feedback variables by the real-time controller. The NMES control algorithm sequentially controls each joint angle while locking the other DoFs.

The user interacts with the system by means of an eyetracker. Therefore, a commercial system, the Tobii T60W system (Tobii Technology AB, Sweden), has been extended by a specific GUI for the MUNDUS application. The table-mounted eyetracker is integrated into a 17″ TFT monitor. During tracking, the Tobii T60 uses infrared diodes to generate reflection patterns on the corneas of the user's eyes. Proper image processing is used to identify the gaze point on the screen. The three dimensional position of the user's hand, of the objects to be manipulated, and of the mouth are continuously monitored by environmental sensors, i.e., two Kinect cameras (Microsoft Corp., Redmond, USA). To this end, colored markers are attached to the hand and the objects. The first Kinect camera provides an image of the working space to the eye-tracking screen. To start an interaction with a specific object, the user has to visually fixate this object on the eyetracker screen for a pre-defined time duration. Once an object is selected, the corresponding Kinect coordinates are sent to the CC which transforms these coordinates into the global (exoskeleton) 3D coordinate system. The transformed coordinates will then be used by the real-time controller for movement generation. The second Kinect camera is placed in front of the user and is used to track the face position.

The fixation detection algorithm has been exclusively developed for the specific MUNDUS application, and it comprises user-dependent temporal (i.e., time during which the user has to continuously fix an object or an icon on the screen to select the gazed point) and spatial (i.e., area around the barycenter of the cluster of gaze samples inside which each sample has to fit for a fixation to be revealed) threshold settings. To prevent unwanted fixation detections, a confirmation icon is shown on the eye-tracking screen after a fixation event is detected, and the user is asked to confirm or cancel the selection. Moreover, the working space where the user can select the object/action to interact with is shown only when the user him/herself has selected the START icon from the standby interface that is provided by the eyetracking screen when MUNDUS is waiting for user interaction.

Special parts of the eye-tracker screen are dedicated to other available tasks (e.g., activating emergency switch off, touching spots of the body). The emergency icon is always displayed in the top-left corner of the screen, and it is continuously selectable to allow the user to stop MUNDUS. If the emergency icon is fixated, a message is sent by the eye-tracker that stops all MUNDUS components. To trigger sub-actions, specific questions are displayed on the screen and the user can reply by fixating a GO or a STOP icon.

The central controller interfaces all modules and interacts with the eyetracker and the real-time controller. For the purpose of system integration, the software components of the CC and the eyetracker module have been integrated in one single MS Windows-based PC. The real-time controller and the data processing of the environmental sensor module are based on a computer system running Linux with RTAI extension^1^. Development and testing of the control system is performed in Scilab/Scicos 4.1.2^2^ using the real-time framework OpenRTDynamics^3^. The communication between all modules is established via UDP and messages are broadcasted in XML format.

### 2.2. Exoskeleton

**Figure 2:**
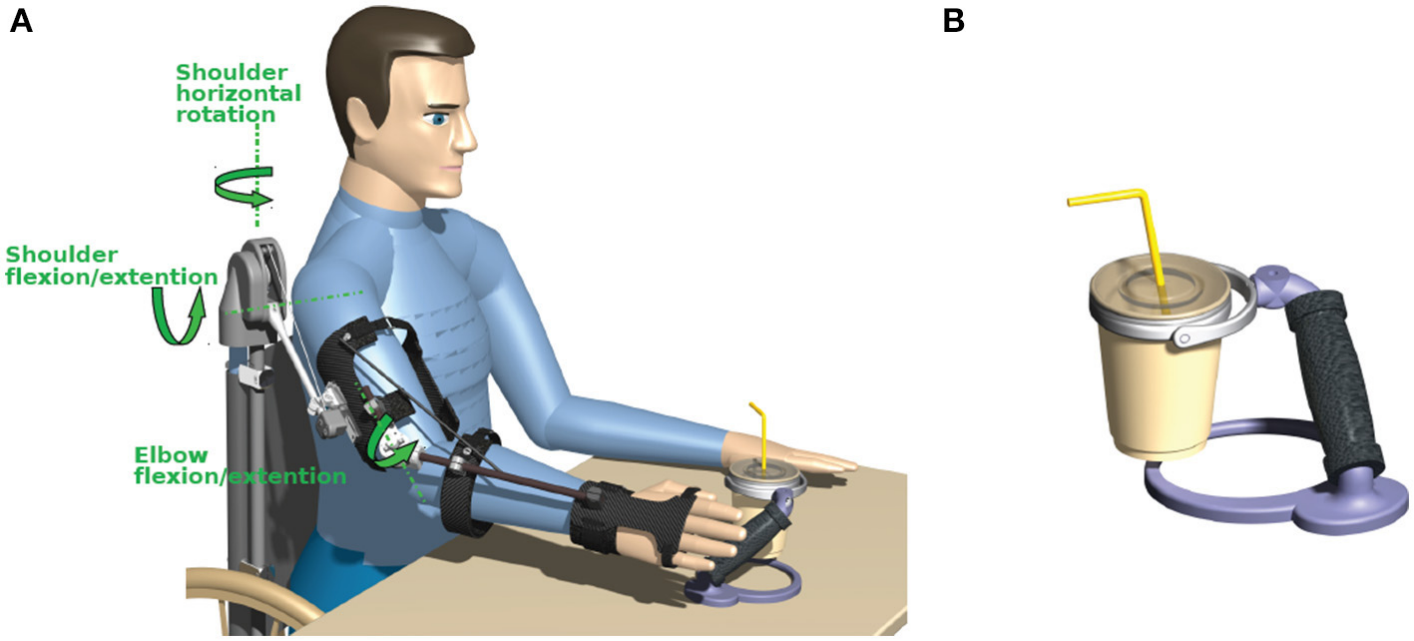
**(A)** Exoskeleton with spring-based gravity compensation and electromagnetic brakes mounted on a wheelchair. **(B)** Cup holder with an universal joint in the handle.

As a basis for the exoskeleton design, the previously mentioned target motions were analyzed using a motion capture system (Lukotronic, Lutz Mechatronic Technology e.U, Austria) to estimate the required ranges of motion and expected loads at the joints (Karner et al., 2012; Reichenfelser et al., 2013). The 3D mechanical design was done in Catia V5R19 (Dassault Systmes, France), focusing on modularity, simplicity and light weight. The developed exoskeleton with gravity compensation is shown in Figure 2A. The available degrees of freedom (DoF) of the exoskeleton are:

1. Shoulder flexion/extension (angle ϑ_*u*_),
2. Shoulder horizontal rotation (angle φ_*u*_),
3. Elbow flexion/extension (angle ϑ_*f*_).

The rotation of the forearm around the upper arm axis (humeral rotation) and pronation/supination of the forearm are locked by the exoskeleton as these DoFs are difficult to be controlled by NMES using surface electrodes. Due to the reduced DoFs, the orientation of the hand is not freely adjustable in the workspace. Thus, to allow a safe handling of objects despite this constraint, special objects with an universal joint in the handle have been developed (e.g., cup holder shown in Figure 2B).

The exoskeleton is equipped with magnetic encoders (Vert-X, Contelec AG, Switzerland) to measure the angles for all three DoFs. Electromagnetic DC brakes (Kendrion, Germany) can lock the shoulder horizontal rotation with a torque of 2.5 Nm, the shoulder flexion/extension with up to 5 Nm and the elbow flexion/extension with 1.5 Nm to hold the arm in any posture when the stimulation is switched off.

**Figure 3:**
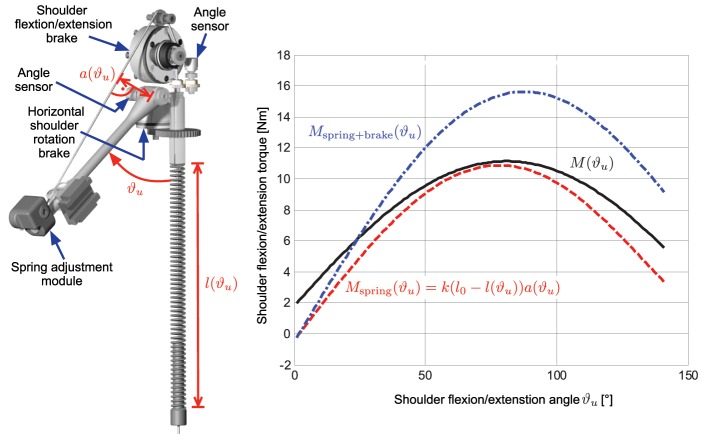
**Isometric view of the shoulder joint mechanism showing the angle sensors and brakes for the two degrees of freedom.** The right graph shows the occurring torque due to gravity (black solid line) together with the compensation torque (dashed red line) at the shoulder joint as a function of shoulder flexion/extension angle ϑ_*u*_ for an averaged upper arm weight of 2.15 kg and a forearm/hand weight of 1.91 kg with the elbow flexed at 90°. The resulting additional torque when the electromagnetic brake is switched on is shown as blue dash-dotted line.

To realize gravity compensation, a pressure spring is integrated in a vertical carbon tube that can be either mounted on a wheelchair as shown in Figure 2 or alternatively attached to a body harness for mobile use. The spring force is transferred to the elevation lever by a rope and pulley mechanism. Figure 3 depicts an isometric view of the shoulder joint mechanism and shows the occurring torques as a function of shoulder elevation angle. A slight under-compensation (spring torque smaller than gravity torque) is intended as the arm should move downwards slowly and gravity-induced when the stimulation and the brakes are turned off. The amount of compensation is adjusted manually by changing the wind up length of the rope at the spring adjustment module. A linear guiding provides the connection between the elevation lever and the upper arm shell and compensates misalignment of the anatomical and the mechanical shoulder joint. This also minimizes the reaction forces. For the elbow-joint, an elastic band with a variable attachment point acts as weight support.

The exoskeleton has a total weight of 2.2 kg and can be quickly adjusted to different anthropometric dimensions.

### 2.3. Neuro-muscular electrical stimulation

The desired arm movements are induced by four stimulation channels activating the anterior, posterior and medial deltoid as well as the biceps muscle (cf. Table 1). By stimulating the medial deltoid, the shoulder extension can be actuated, while the anterior and posterior deltoid allow arm rotation in the horizontal plane. Stimulation of the biceps is used to flex the elbow-joint. Shoulder flexion as well as elbow extension are induced by gravitational forces.

**Table 1:** **Stimulation channels**.

**Channel**	**Activated muscle**	**Control signal**	**Actuated angle—movement**
1	Biceps	ν_b_	ϑ_*f*_—elbow flexion/extension
2	Deltoid, anterior head	ν_*d*,*a*_	Positive direction of φ_*u*_—shoulder horizontal rotation
3	Deltoid, posterior head	ν_*d*,*p*_	Negative direction of φ_*u*_—shoulder horizontal rotation
4	Deltoid, medial head	ν_*d*,*m*_	ϑ_*u*_—shoulder flexion/extension

One pair of self-adhesive hydrogel electrodes (oval shaped with size 4 × 6.4 cm) is used for each stimulated muscle. For the generation of the biphasic stimulation pulses, the current-controlled stimulator RehaStim Pro (HASOMED GmbH, Germany) is used. The stimulation frequency for all channels is fixed at 25 Hz, while the individual current amplitudes and pulse widths can be adjusted in real-time using the open ScienceMode protocol^4^ through a galvanically isolated USB interface.

The stimulation intensity in terms of pulse charge ν_*i*_ serves as control signal for the muscle *i*. Table 1 shows the used control signal notation. The pulse charge ν_*i*_ of the muscle *i* is defined as product of the current amplitude *I*_*i*_ and the pulsewidth *pw*_*i*_. In this application, a given charge is equally distributed to pulse width and current amplitude (normalized to their maximal values) as follows:

$$pw_{i}=\sqrt{\frac{\nu_{i}pw_{\text{max}}}{I_{\text{max}}}},\;I_{i}=\sqrt{\frac{\nu_{i}I_{\text{max}}}{pw_{\text{max}}}},\;0\leq \nu_{i}\leq (I_{\text{max}}pw_{\text{max}}),$$

where *pw*_max_ = 500 μs and *I*_max_ = 127 mA are the maximal values of pulse width and current amplitude, respectively.

In a calibration phase that is always performed before using the MUNDUS system, the maximal tolerated pulse charge ν_*i*_ of each muscle *i* is determined. Additionally, for the medial deltoid, the stimulation intensity ν_*d*,*m*_ that causes the onset of a visible muscle contraction is determined. This value is required for the implementation of the more complex shoulder flexion/extension controller described in Section 2.5.2.

### 2.4. Kinematic model and coordinate transformations

To calculate the hand position from a given set of joint angles or vice versa, a kinematic model of the exoskeleton is required. In addition, a transformation from the Kinect coordinate system to the global (exoskeleton) coordinate system must be determined for the following reason: Objects to interact with may be arbitrarily located on the table in front of the user. The Kinect is required to determine the object position in the local Kinect coordinate system. In order to bring the hand to objects by NMES, the Kinect coordinates must be mapped into exoskeleton 3D coordinates and corresponding exoskeleton angles. The latter are used to describe the hand position in the real-time arm controller.

It is assumed that the placement of the Kinect as well as the settings of the exoskeleton may change from day to day. Therefore parameters need to be determined with simple and fast procedure through experimental system identification.

**Figure 4:**
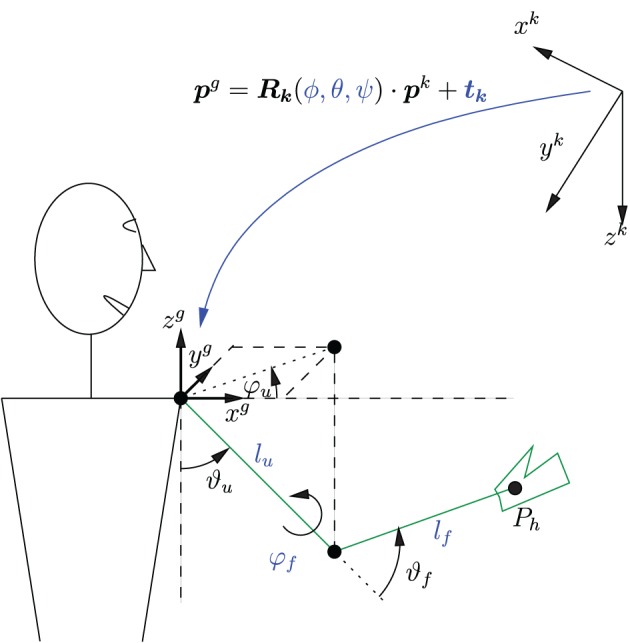
**Simplified kinematic model of the exoskeleton with coordinate systems and a transformation between these systems.** Depicted is the right arm reaching forward. The parameters of the coordinate transformation ϕ, θ, ψ, and ***t**_k_* as well as the kinematic model parameters *l_u_*, *l_f_*, and φ_*f*_ need to be identified.

Figure 4 shows the simplified kinematic exoskeleton/arm model with the global (exoskeleton) coordinate system (*x^g^*, *y^g^*, *z^g^*) and the Kinect coordinate system (*x^k^*, *y^k^*, *z^k^*). Both are Cartesian coordinate systems. Depicted is the right arm reaching forward. The model assumes that the exoskeleton is completely rigid and that the arm is perfectly aligned to the exoskeleton.

The forward kinematics is given by

(1) $$p_{h}^{g}(\vartheta_{u},\varphi_{u},\vartheta_{f})\;=-(l_{u}R(\vartheta_{u},\varphi_{u})+l_{f}R(\vartheta_{u},\varphi_{u})R(\vartheta_{f},\varphi_{f}))e_{z}.$$

where ***p^g^_h_*** is the hand position in global coordinates, ***e_z_*** = [0, 0, 1]*^T^* is a unity vector, and *l_f_* and *l_u_* are the lengths of the forearm and upper arm, respectively. The rotation matrix ***R*** is defined as follows:

(2) $$R(\vartheta ,\varphi ):=\left[\begin{array}{ccc} \cos\varphi \cos\vartheta & -\sin\varphi & -\sin\vartheta \cos\varphi \\ \cos\vartheta \sin\varphi & \cos\varphi & -\sin\varphi \sin\vartheta \\ \sin\vartheta & 0 & \cos\vartheta \end{array}\right].$$

In the used setup, the humeral rotation angle φ_*f*_ of the shoulder is constant, as it represents a fixed DoF, and its value is determined by the configuration of the exoskeleton.

Equation (1) can be used to determine the hand position for a given set of exoskeleton angles. The *inverse kinematics* can be obtained by numerically solving Equation (1) to determine the angles ϑ_*u*_, φ_*u*_ and ϑ_*f*_ for a given hand position ***p^g^_h_*** within the reachable workspace and angle φ_*f*_. The solution is unique as the humeral shoulder rotation angle φ_*f*_ is fixed, and the operational space for ϑ_*f*_ is limited by the mechanical constraints to [0, π].

The transformation from Kinect coordinates to global coordinates is visualized in Figure 4 and can be written as

(3) $$p^{g}\;=\;R_{k}(\phi ,\;\theta ,\psi )p^{k}+\;t_{k}$$

where ***p^g^*** = [*x^g^ y^g^ z^g^*]^*T*^, ***p^k^*** = [*x^k^ y^k^ z^k^*]^*T*^, and ***t_k_*** ∈ ℝ^3 × 1^ is a translation vector, and ***R_k_*** ∈ ℝ^3 × 3^ a rotation matrix which is parameterized by the Euler angles ϕ, θ, and ψ.

#### 2.4.1. Parameter identification

The parameters ϕ, θ, ψ, and ***t_k_*** of the coordinate transformation as well as the kinematic model parameters *l_u_*, *l_f_*, and φ_*f*_ are unknown and have to be calibrated for each user each time the system is set up. Therefore, a system identification procedure is applied to determine the nine parameters. During the calibration phase, the arm and the attached unlocked exoskeleton are manually placed by a third person (e.g., the caregiver) at *N* different positions in the reachable workspace that can be reached with the arm attached to the exoskeleton. Since nine parameters need to be identified, *N* ≥ 9 positions must be visited. The reachable workspace is at first defined by the forward kinematics of the exoskeleton. However, this space may be furthermore limited by insufficient NMES-induced muscle force.

For each hand position *i*, the corresponding joint angles (ϑ_*u*,*i*_, φ_*u*,*i*_, ϑ_*f*,*i*_) are measured together with the hand position vector

(4) $$p_{h,i}^{k}={\left[\begin{array}{ccc} x_{h,i}^{k} & y_{h,i}^{k} & z_{h,i}^{k} \end{array}\right]}^{T},$$

which is recorded by the environmental sensor in the Kinect coordinate frame.

The unknown parameter vector **Θ** = [*l_u_ l_f_* φ_*f*_ ϕ θ ψ ***t_k_**^T^*]*^T^* is estimated by minimizing a quadratic cost function

(5) $$\hat{\Theta }=\arg\underset{\Theta }{\min}\left(\frac{1}{2}\sum_{i\;=\;1}^{N}e_{i}e_{i}^{T}\right)$$

where

(6) $$\begin{array}{l} e_{i}\;:\;=\underset{p_{h,i,\text{FK}}^{g}}{\underset{︸}{\left(-(l_{u}R(\vartheta_{u,i},\varphi_{u,i})+l_{f}R(\vartheta_{u,i},\varphi_{u,i})R(\vartheta_{f,i},\varphi_{f}))\;e_{z}\right)}}\\ \;\underset{p_{h,i,\text{Kinect}}^{g}}{\underset{︸}{-(R_{k}(\phi ,\;\theta ,\psi )\cdot \;p_{h,i}^{k}+\;t_{k})}} \end{array}$$

is the error between the hand position ***p**^g^*_*h*,*i*,FK_, obtained by the forward kinematic model (1), and the hand position ***p**^g^*_*h*,*i*,Kinect_, obtained from the transformed Kinect measurements, both in global coordinates. The minimization of the cost function is achieved by the Gauss-Newton method with analytically calculated gradients.

### 2.5. Control system

All NMES generated arm movements are initiated by commands received from the high level control system, the Central Controller (CC), which processes, among others, the information collected by the eye-tracker. The CC movement commands are:

1. Move hand to a desired 3D position,
2. Change the angle of shoulder flexion/extension by a certain amount, and
3. Change the angle of elbow flexion/extension by a certain amount.

Each command emits an event causing a state transition in a finite state-machine on the real-time control system, which then performs the actual movement.

**Figure 5:**
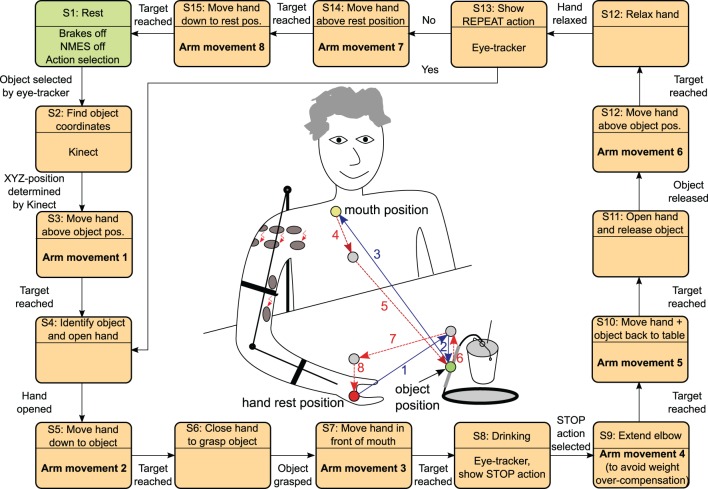
**The state automaton inside the MUNDUS Central Controller (CC) to realize the drinking use case starting from an arm rest position and returning to this position again.** The states (S3, S5, S7, S9, S10, S12, S14, S15) with arm movements trigger a state machine inside the real-time arm NMES control module (cf. Figure 6). The references for the rest position as well as for the mouth position may be stored in the MUNDUS CC as angular references during the system calibration phase. The object position is online determined by the Kinect system by tracking a green marker on the object handle.

Based on the elementary movement commands outlined above, complex movement sequences are possible by a combination of multiple commands issued in series. An example for the drinking use case is outlined in Figure 5.

In this study, the hand movements were performed voluntarily by the subject. In the complete MUNDUS system, two alternative solutions to support hand functions have been proposed: a hand neuroprosthesis and a robotic hand orthosis (Pedrocchi et al., 2013). The hand neuroprosthesis deploys a new stimulation system for array electrodes (Valtin et al., 2012) in order to produce precise finger movements. However, the description of these hand modules is outside the scope of this study.

It should be noted that the straight lines shown in the center of Figure 5 do not represent the actual trajectories of the hand. The actual generation of a movement between two points by the real-time controller will be described in the next section.

#### 2.5.1. Sequential real-time control strategy

The real-time control system internally controls the angles of the exoskeleton. Therefore, whenever a command is issued by the CC, new angular references are determined by the real-time control system. This calculation involves, if required, also stored old angular references from the last movement and the inverse exoskeleton kinematics. The resulting reference angles of the *j*th command are *r^j^*_ϑ_*u*__, *r^j^*_φ_*u*__, and *r^j^*_ϑ_*f*__ for the shoulder ab-/adduction, the horizontal shoulder rotation, and the elbow flexion/extension, respectively.

Sequential feedback control is used to adjust the stimulation intensities (pulse charges) in order to drive the hand to desired positions in the reachable work space. Each DoF is controlled separately, one after the other while all other DoFs are locked by the exoskeleton brakes. This results in a fully decoupled system with regard to crosstalk between the DoFs. For this reason, a light model with few parameters can be used for each controller design, which dramatically reduces the effort for parameter identification. Each movement to a given 3d position is divided into three consecutive steps:

1. control of the shoulder flexion/extension,
2. control of the shoulder horizontal rotation and
3. control of the elbow flexion/extension.

**Figure 6:**
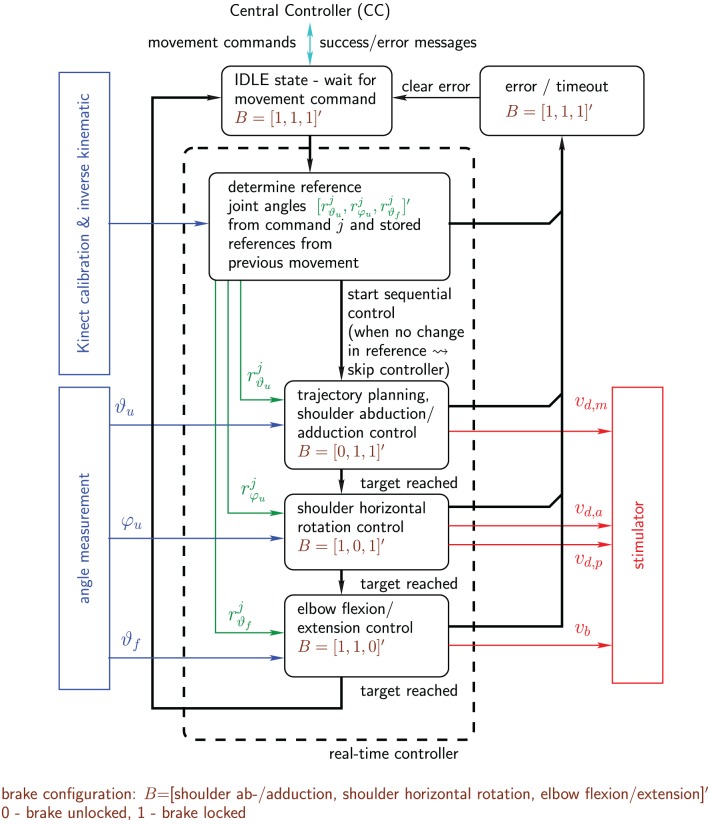
**Real-time arm NMES control system shown in form of a hybrid system combining a state automaton and continuous controllers: state transitions are indicated by black bold arrows, while continuous signals are represented by colored thin arrows.** Not shown are short periods (states) between the activations of the individual controllers in which all brakes are locked and the respective initial stimulation intensities are adjusted for the next controller activation.

The real-time arm NMES controller is a hybrid control system combining a state automaton and continuous-time feedback controllers to reach the desired angle subsequently for each DOF (cf. Figure 6).

#### 2.5.2. Shoulder flexion/extension control

For the shoulder flexion/extension, a discrete-time controller based on an identified pulse transfer-function model is employed. The control design uses the well-known pole-placement method in polynomial form (Astrom and Wittenmark, 1996). For the *j*th activation of the controller, the relation between the stimulation intensity ν^*j*^_*d*,*m*_ of medial deltoid and the shoulder elevation angle ϑ*^j^_u_* can be approximately described by a second order *autoregressive with exogenous input* (ARX) model (Ljung, 1999) of the form

(7) $$\begin{array}{l} \vartheta_{u}^{j}(k)\;=\frac{B(q)}{A(q)}\nu_{d,m}^{\;j}(k)+\frac{q^{2}}{A(q)}e^{\;j}(k),\\ \;{\underline{v}}_{d,m}\leq \nu_{d,m}^{\;j}(k)\leq {\bar{\nu }}_{d,m},k\geq 0, \end{array}$$

where *k* is the sample index, *e^j^*(*k*) represents white noise, and

$$\begin{array}{l} B(q)=b_{0},\\ A(q)=(q^{2}+a_{1}q+a_{2})q^{4} \end{array}$$

are polynomials of the forward-shift operator *q* (*q* *s*(*k*) = *s*(*k* + 1)). This model possesses an input-output time delay of six sampling instants, which is typically observed in the recorded I/O data. The used sampling frequency is 25 Hz and equals to the stimulation frequency. During the system calibration, the coefficients of the polynomials are estimated from a recorded input step response (changing ν_*d*,*m*_ from (ν_*d*,*m*_ + 0.2(ν_*d*,*m*_ − ν_*d*,*m*_)) to (ν_*d*,*m*_ + 0.8(ν_*d*,*m*_ − ν_*d*,*m*_))) using the instrumental variable method (Ljung, 1999).

**Figure 7:**
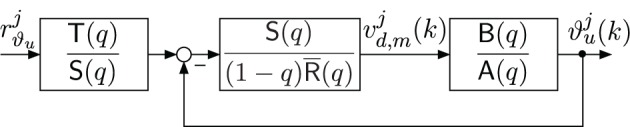
**Closed-loop system with discrete-time polynomial controller**.

Based on the obtained model, a polynomial controller of the form

(8) $$\nu_{d,m}^{j}(k)=\frac{S(q)}{\bar{R}(q)(1-q)}\left(\frac{T(q)}{S(q)}r_{\vartheta_{u}}^{j}-\vartheta_{u}^{j}(k)\right)$$

is designed with the controller polynomials R(*q*), S(*q*), and T(*q*). Figure 7 shows the corresponding closed-loop system. The controller has integral action [factor (1 − q) in (8)]. This enables the rejection of constant and slowly varying disturbances and compensates the effects of muscular fatigue. The coefficients of the controller polynomials R(*q*) and S(*q*) are chosen to obtain a desired characteristic polynomial

(9) $$A_{cl}(q)=(1-q)\bar{R}(q)A(q)+S(q)B(q)$$

the roots of which are equal to the closed-loop system poles and should be stable and well damped. For the given system and controller with integrator, the minimal degree controller is given by deg(S) = 6, R) = 5 and deg A_*cl*_ = 12. A common approach is to factorize A_*cl*_(*q*) as follows:

(10) $$A_{cl}(q)=A_{cl,1}(q)A_{cl,2}(q)q^{8}$$

where A_*cl*,1_(*q*) and A_*cl*,2_(*q*) are second order polynomials specified via rise-time *t_r,i_* and damping factor *D*_*i*_ (*i* = 1,2) of corresponding continuous-time second order systems. Eight of the twelve closed-loop poles are located at the origin (fastest possible mode in discrete-time). The pre-filter polynomial is set to

(11) $$T(q)=\;A_{cl,2}(q)q^{4}A_{cl,1}(1)/B(1).$$

This yields a unity DC gain from the reference input *r^j^*_ϑ_*u*__ to the system output ϑ*^j^_u_*. Furthermore, it cancels six closed-loop poles defined by A_*cl*,2_(*q*)*q*^4^. The resulting transfer function of the closed-loop system is then:

(12) $$\frac{\vartheta_{u}^{j}(k)}{r_{\vartheta_{u}}^{j}(k)}=\frac{T(q)B(q)}{A_{cl}(q)}=\frac{A_{cl,1}(1)B(q)}{q^{4}A_{cl,1}(q)B(1)}.$$

As a result, only the poles defined by the roots of *q*^4^A_*cl*,1_(*q*) influence the system dynamics with respect to changes in the reference signal. The disturbance rejection and noise properties of the closed-loop system, however, are depending on all closed-loop poles defined by Equation (10). At first, the rise-time and damping factor for A_*cl*,1_ are selected to obtain a desired reference tracking behavior. Then the rise-time and damping factor of A_*cl*,2_ are iteratively tuned to yield satisfactory noise sensitivity and disturbance rejection (verified by frequency response plots of the sensitivity and the complementary sensitivity function). For all subjects of this study, we have chosen *t*_*r*,1_ = 0.6 s, *t*_*r*,2_ = 0.5 s and a damping factor *D*_*i*_ = 0.999 for both polynomials.

**Figure 8:**
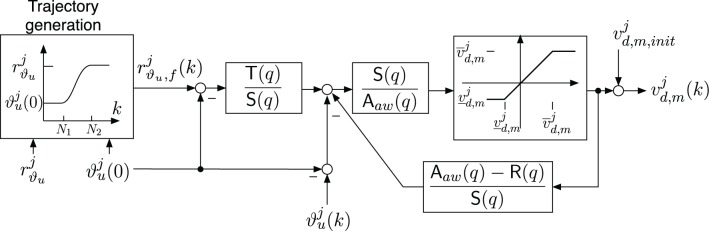
**Implementation of the shoulder extension/flexion controller including an anti-windup observer with R(*q*) = (1 − *q*) R(*q*), a trajectory generator and an adjustable initial stimulation intensity ν*^j^_d,m,init_*.** The parameters of the saturation function are ν*^j^*_*d*,*m*_ = ν_*d*,*m*_- ν*^j^_d,m,init_* and ν*^j^*_*d*,*m*_ = ν_*d*,*m*_-ν*^j^_d,m,init_* for ν_*d*,*m*_ ≤ ν*^j^_d,m,init_* ≤ ν_*d*,*m*_.

The final controller implementation, which is shown in Figure 8, takes the following additional aspects into account:

1. Controller initialization to apply a given constant initial stimulation intensity ν*^j^_d, m_*(0) = ν*^j^_d, m, init_*.
2. Generation of a smooth reference trajectory *r^j^*_ϑ_*u*_,*f*_(*k*) that guides the arm from the initially measured angle ϑ*^j^_u_*(0) to the given target angle *r^j^*_ϑ_*u*__ of the activation *j*.
3. Avoidance of integrator windup for control signals violating the constraint ν_*d*,*m*_ ≤ ν^*j*^_*d*,*m*_(*k*) ≤ ν_*d*,*m*_ by using the standard anti-windup scheme proposed in Astrom and Wittenmark (1996) with the anti-windup observer polynomial A_*aw*_(*q*) = A_*cl*,2_(*q*)*q*^4^.

The initial stimulation intensity ν*^j^_d,m,init_* is adjusted in order to avoid undesired movements when the controller is activated. Thus, before the controller activation and the brake release, the stimulation intensity is increased up to the value which was used before locking the DoF. The ramp-up period lasts about 1.5 s. Furthermore, to avoid unwanted initial transients caused by the controller transfer functions, the initial joint angle ϑ*^j^_u_*(*k* = 0) at controller activation is acquired and then subtracted from the joint angle measurement ϑ*^j^_u_*(*k*) and the output of the trajectory generator.

#### 2.5.3. Trajectory generation

To obtain smooth shoulder flexion/extension movements, the reference trajectory *r^j^*_ϑ_*u*_,*f*_(*k*) for each activation *j* is chosen to be a sinusoidal reference path starting at ϑ*^j^_u_*(0) and converging to the desired target angle *r^j^*_ϑ_*u*__:

$$r_{\vartheta_{u},f}^{j}(k)=\{\begin{array}{ccc} \vartheta_{u}^{j}(0) & \text{ for} & 0\leq k<N_{1}\\ \begin{array}{c} \frac{1}{2}\left(1-\cos\left(\frac{\pi k-N_{1}}{2N}\right)\right)\cdot \\ \left(r_{\vartheta_{u}}^{j}-\vartheta_{u}^{j}(0)\right)+\vartheta_{u}^{j}(0) \end{array} & \text{ for} & N_{1}\leq k\leq N_{2}=N_{1}+N\\ r_{\vartheta_{u}}^{j} & \text{ for} & k>N_{2}=N_{1}+N \end{array}\text{​​​}.$$

The parameter *N*_1_ = 69 describes the amount of samples (corresponding to 2.76 s) before the sinusoidal shape starts, and *N* denotes the number of samples for the transient part of the trajectory and is set to 150 (corresponding to 3 s). After the sample *N*_2_ = *N*_1_ + *N*, the reference trajectory is equal to *r^j^*_ϑ_*u*__. Then, the controller will be deactivated and the brake will be locked as soon as one of the following conditions is fulfilled:

- The absolute error |*r^j^*_ϑ_*u*__ − ϑ*^j^_u_*(*k*)| is less than 1°.
- The control signal ν^*j*^_*d*,*m*_(*k*) was continuously saturated for more than 2 s.
- The controller was active for more than 15 s (*time-out event*).

Once the target is reached, the current value of stimulation intensity is stored and the controller of the shoulder flexion/extension is deactivated.

#### 2.5.4. Shoulder horizontal rotation control

The control of the shoulder horizontal rotation involves the stimulation of the anterior (for inward rotation) and the posterior (for outward rotation) deltoid. Thus, the following switching control law is used

(13) $$\nu_{d,a}^{j}=\{\begin{array}{ccc} u_{r}^{j} & \text{ if} & u_{r}^{j}>0\\ 0 & \text{ if} & u_{r}^{j}\leq 0 \end{array}$$

(14) $$\nu_{d,p}^{j}=\{\begin{array}{ccc} -u_{r}^{j} & \text{ if} & u_{r}^{j}<0\\ 0 & \text{ if} & u_{r}^{j}\geq 0 \end{array},$$

which introduces a mapping of one single virtual actuation variable *u^j^_r_* ∈ [−ν_*d*,*p*_,ν_*d*,*a*_] to the two stimulation intensities ν^*j*^_*d*,*a*_ and ν^*j*^_*d*,*p*_ for the *j*th controller activation.

The virtual actuation variable *u^j^_r_* is the output of an integral controller with constant integration slopes and is given by

$$u_{r}^{j}(k+1)=\underset{-{\bar{\nu }}_{d,p},{\bar{\nu }}_{d,a}}{\text{sat}}\left(u_{r}^{j}(k)+c_{r}\text{sgn}(r_{\varphi_{u}}^{j}-\varphi_{u}^{j}(k))\right),\;u_{r}^{j}(0)=0,$$

where the positive gain *c_r_* is set to 0.3 μ as in this study. To avoid integrator windup, a saturation function

(15) $$\underset{b_{1},b_{2}}{\text{sat}}(x):=\{\begin{array}{ccc} b_{1} & \text{ if} & x\leq b_{1}\\ x & \text{ if} & b_{1}<x<b_{2}\\ b_{2} & \text{ if} & b_{2}\leq x \end{array}$$

is used in the integral control law. This prevents the integrator from exceeding the constraints for the actuation variable.

Conditions for the deactivation of the controller and the subsequent locking of the brake are in analogy to the ones given in Section 2.5.2.

#### 2.5.5. Elbow extension/flexion control

The control of elbow extension/flexion is similar to the horizontal shoulder rotation control, but only one muscle, the biceps, is stimulated in order to induce elbow flexion. Downward movements of the forearm (extensive movements) are caused by gravity. The stimulation intensity will be linearly increased/decreased with the absolute slope rate *c_e_* = 6.7 nAs in each sampling instance until the desired angle is achieved. The following integral controller, which also includes an anti-windup strategy, is used:

(16) $$\nu_{b}^{j}(k+1)=\underset{0,{\bar{\nu }}_{b}}{\text{sat}}\left(\nu_{b}^{j}(k)+c_{e}\text{sgn}\left(r_{\vartheta_{f}}^{j}-\vartheta_{f}^{j}(k)\right)\right),\;\nu_{b}^{j}(0)=\nu_{b,init}^{j}.$$

Here, *j* represents again the *j*th activation of the controller. The initial stimulation intensity ν*^j^_b,init_* is adjusted in order to prevent the forearm from rapidly falling down when the controller is activated and the brake is released. Thus, before the controller activation, the stimulation intensity is increased up to 50% of the stimulation intensity achieved at the end of the previous activation phase of the elbow controller. The ramp-up phase lasts 1 s.

Conditions for the deactivation of the controller and the subsequent locking of the brake are in analogy to the ones given in Section 2.5.2.

### 2.6. Validation of the control system

The control system was validated in five healthy subjects (three female and two male), aged 29–40 years (mean ± *SD* 34.5 ± 5.3). Average weight was 61 ± 17 kg. The drinking task was selected to evaluate the performance of the system. Each subject was asked to be completely relaxed during the arm movements entirely induced by the system. At the hand related steps of the procedure, he/she was asked to voluntarily open and close the hand in order to grasp and release the cup. Each subject repeated the trial five times. Before the beginning of the trials, the exoskeleton as well as the amount of gravity compensation were adjusted to the anthropometric measures of each subject. Then, the system was calibrated performing the following steps:

- Set the stimulation parameters (Section 2.3),
- Determine the parameters of the kinematic model and coordinate transformation (Section 2.4),
- Tune the discrete-time controller of the shoulder flexion/extention by means of an experimental session aimed at model identification (Section 2.5), and
- Teach-in the rest position and the in-front-of-mouth position.

The experimental protocol was approved by the ethical committee of the Valduce Hospital (Italy) where the validation trials have been performed. All subjects signed a written informed consent.

To evaluate the performance of the system, the positioning error between the target position and actually reached position at the completion of each movement command was computed for the hand positions 1 to 8 shown in Figure 5. Two sets of positioning errors were calculated since two different methods were used to derive the actual position in the global coordinate system: (1) the measured angles were applied to the forward kinematic model; (2) the actual position measured by the Kinect was transformed in the global coordinate system. Furthermore, the time needed to execute all movement commands during the drinking task was computed.

## 3. Results

**Figure 9:**
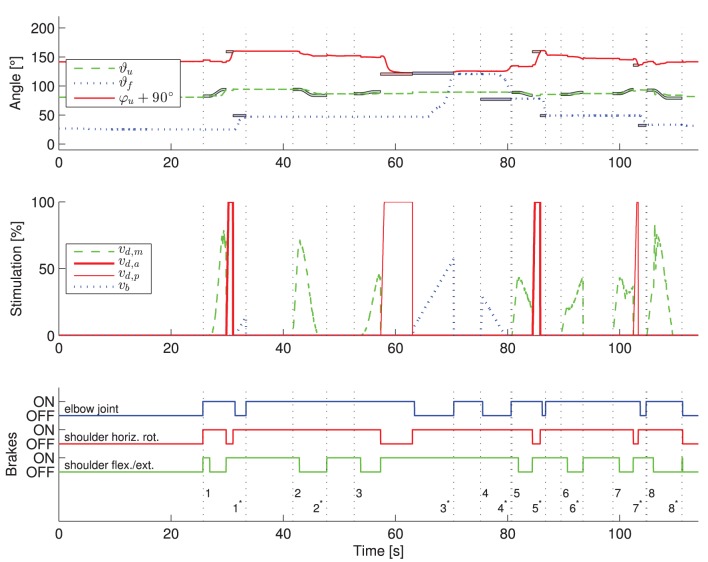
**Exemplary results of the application of the developed control system to one healthy subject.** The transient behavior for one trial of the described drinking task is shown. The numbers on the vertical dashed lines in the third subplot indicate the begin (without star) and end (with star) of the eight arm movements defined in Figure 5. In the first subplot, the active reference angles (bold colored lines with black surrounding) are shown along with the measured angles. In the figure, the colors blue, green and red correspond to the elbow-joint, shoulder flexion/extension and shoulder horizontal rotation, respectively. In the middle subplot, the applied stimulation intensities are presented. The state of the brakes is plotted in the bottom subplot. An individual controller for one DOF is only active for time periods in which a reference trajectory is plotted for the corresponding angle. Theoretically, angles should not change in periods in which no corresponding reference trajectories are plotted due to active brakes.

**Figure 10:**
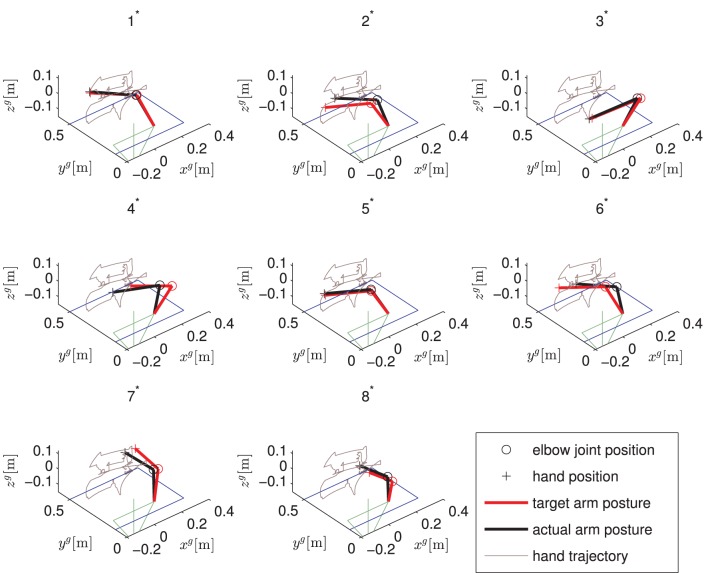
**Static arm postures for one trial of the described drinking task.** Shown are the desired arm postures and the actually obtained ones for the endings of the eight arm movements defined in Figure 5. The upper body is indicated in green while the right arm is pointing forwards. The table in front of the subject is illustrated in blue.

Figure 9 exemplarily shows the recorded angles together with their active references (bands), the applied stimulation intensities and the states of the brakes. Vertical, dashed lines separate the time periods of the controlled arm movements that have been introduced and numbered in Figure 5. The stimulation intensities ν_*d*,*a*_, ν_*d*,*m*_, ν_*d*,*p*_, and ν_b_ are normalized to their bounds [0,ν_*d*,*a*_], [ν_*d*,*m*_,ν_*d*,*m*_], [0,ν_*d*,*p*_], and [0,ν_b_], respectively. The control system is performing well in moving the arm such that the joint angles are close to the reference angles. However, in this example, an unwanted slipping of the horizontal shoulder brake can be observed after 43, 80, 92, and 106 s that causes the shoulder horizontal rotation angle φ_*u*_ to drift away from the previously reached target angle. Figure 10 shows the desired arm posture at the ending of every controlled arm movement in comparison to the real arm position achieved by NMES. The error caused by slipping is clearly visible for the instances of time 2^*^, 4^*^, 6^*^, and 7^*^, which represent the endings of the corresponding movements defined in Figure 5.

The five trials of the drinking task were successfully completed by all subjects. For each subject, Table 2 reports the mean and standard deviation values of the position errors in *x*^g^/*y^g^*/*z^g^*-directions obtained during the five trials of the drinking task. The controller performance obtained in the two most important reaching subactions, i.e., reaching the object and reaching the mouth, and the overall performance obtained by averaging the results obtained in all of the eight target positions are shown in Table 2. The Euclidian norm (i.e., the mean distance error) of the mean positioning error vectors has been calculated from data in Table 2 and is reported in Table 3. The mean distance error for all subjects and positions was less than two centimeters when using the exoskeleton angles to determine the hand position. Based on the Kinect measurements, the observed mean distance error is smaller than five centimeters. For the majority of subjects (B–E), a relatively large mean (systematic) error in the *x^g^*-direction of up to 12 cm are observed for the object position (cf. Figure 5), resulting in a mean distance of about 8 cm (see Table 3). Subject D obtained a large standard deviation for the object positioning error in *x^g^*-direction (see Table 2). A larger discrepancy between the errors based on the exoskeleton sensors and the Kinect can be observed for the mouth position in subjects C–E.

**Table 2:** **Mean positioning errors along with their standard deviations in *x^g^*/*y^g^*/*z^g^*-direction for five drinking task sequences per subject measured via the exoskeleton sensors and via Kinect**.

**Subject (Healthy)**	**RMS [cm] error of Kinematic model calibration**	**Mean positioning errors (*SD*) in *x^g^*/*y^g^*/*z^g^*-direction [cm]**					
		**All positions**		**Mouth**		**Object**	
		**Via exo**	**Via Kinect**	**Via exo**	**Via Kinect**	**Via exo**	**Via Kinect**
A	0.4	0.4 (1.8)/	1.0 (2.2)/	−0.3 (0.5)/	1.3 (0.6)/	−0.4 (0.9)/	0.0 (0.7)/
		−0.1 (0.8)/	−0.0 (1.2)/	−1.3 (0.2)/	−0.6 (1.0)/	−0.8 (0.5)/	−0.9 (0.7)/
		−0.1 (2.1)	1.4 (2.5)	1.0 (0.3)	0.8 (1.0)	−2.5 (1.6)	−1.1 (0.9)
B	1.8	0.6 (7.9)/	−1.4 (6.7)/	−4.8 (5.9)/	−0.38 (1.3)/	−5.5 (5.1)/	−5.6 (2.1)/
		1.8 (4.8)/	3.0 (4.3)/	−0.1 (1.2)/	1.0 (1.3)/	0.4 (0.7)/	2.1 (0.6)/
		−0.2 (3.2)	2.0 (3.9)	0.8 (2.0)	−0.1 (5.0)	1.3 (2.0)	5.1 (2.8)
C	1.4	−1.4 (9.7)/	−3.2 (9.1)/	1.3 (1.2)/	−5.0 (1.0)/	−9.6 (2.1)/	−8.6 (1.4)/
		1.3 (3.9)/	1.5 (3.9)/	−0.6 (0.2)/	1.2 (1.4)/	1.8 (1.4)/	2.0 (1.1)/
		−2.1 (3.6)	1.4 (3.9)	−0.3 (0.3)	−3.0 (1.0)	−1.3 (3.8)	2.0 (2.0)
D	1.4	−0.1 (4.8)/	−0.7 (5.4)/	−0.4 (0.3)/	−3.4 (0.6)/	−6.3 (10.0)/	−6.5 (9.2)/
		−0.6 (1.4)/	−1.5 (3.5)/	−2.1 (0.4)/	−8.9 (0.9/)	−0.8 (0.4)/	0.1 (0.3)/
		−0.4 (1.9)	4.5 (3.3)	0.2 (0.3)	4.2 (0.3)	−2.3 (0.2)	1.3 (0.6)
E	1.7	−1.0 (6.9)/	−2.8 (5.1)/	2.5 (1.3)/	−5.5 (1.5)/	−12.6 (0.5)/	−7.0 (3.1)/
		1.3 (4.0)/	2.4 (3.8)/	−1.1 (0.2)/	−0.9 (0.3)/	1.3 (0.2)/	2.5 (0.4)/
		0.6 (2.6)	3.2 (4.0)	−0.7 (0.1)	−3.7 (0.1)	2.1 (0.3)	5.1 (0.5)

**Table 3:** **Euclidean norm (distance) of the mean positioning error vector given in Table 2**.

**Subject (healthy)**	**Euclidean norm of the mean positioning error vector [cm]**					
	**All positions**		**Mouth**		**Object**	
	**Via exo**	**Via Kinect**	**Via exo**	**Via Kinect**	**Via exo**	**Via Kinect**
A	0.4	1.7	1.7	1.7	2.7	1.4
B	1.9	3.8	4.9	3.9	5.6	7.8
C	2.8	3.8	1.5	5.9	9.9	9.1
D	0.8	4.8	2.1	10.4	6.8	6.7
E	1.8	4.9	2.8	6.7	12.9	9.0
Mean (*SD*)	1.5 (1.0)	3.8 (1.3)	2.6 (1.4)	5.7 (3.3)	7.6 (3.9)	6.8 (3.2)

Additionally to positioning error analysis, the validity of the identified kinematic model and coordinate transformation is investigated for each individual subject. For the twelve positions chosen during the kinematic model calibration, we calculated the 3D position of the hand in two ways using the found kinematic model parameters: At first by applying the kinematic model to the measured exoskeleton joint angles and second by transforming the Kinect measurements into the global coordinate system. Then, over all twelve positions the RMS of the distance error between the two estimates for the hand positions is calculated. The results are shown in Table 2.

The mean values averaged over five trials of the observed time durations for all sub movements and for each subject are reported in Table 4. Each individual sub movement is indicated by a number previously introduced in Figure 5. Additionally, the mean values for the total time required to complete a full drinking task (only time durations wherein the controller was activated are counted) are reported per subject. The average time for the execution of all eight arm movement commands was 71.4 s. The total time for donning the system on and for calibration was less than 10 min for every subject (calibration alone required about 2 min).

**Table 4:** **Mean time durations along with their standard deviations for each sub movement defined in Figure 5 and each subject**.

**Sub movement**	**Mean time durations (SD) [s] for the subjects A–E**					**Mean [s]**
	**A**	**B**	**C**	**D**	**E**	
1	11.2 (0.16)	7.8 (0.22)	13.1 (1.08)	8.4 (0.17)	9.2 (0.27)	9.9
2	7.5 (0.24)	6.0 (0.35)	4.5 (0.05)	8.5 (0.50)	6.0 (0.11)	6.5
3	12.2 (0.39)	11.9 (1.26)	15.6 (0.60)	11.7 (0.14)	16.8 (0.90)	13.6
4	2.3 (0.05)	12.5 (0.49)	5.2 (0.79)	1.7 (0.04)	10.1 (0.40)	6.4
5	10.1 (0.76)	9.6 (0.51)	12.1 (0.71)	13.0 (0.81)	10.1 (0.73)	11.0
6	7.9 (0.57)	3.1 (0.93)	4.9 (0.51)	5.5 (0.29)	3.7 (0.25)	5.0
7	8.2 (0.20)	12.2 (0.61)	9.6 (0.57)	9.4 (0.22)	10.8 (0.91)	10.0
8	9.1 (0.42)	9.7 (0.61)	7.9 (0.15)	6.6 (0.29)	11.6 (0.78)	9.0
Mean of total time duration (*SD*) for five trials [s]	68.3 (2.3)	72.8 (7.8)	73.1 (13.5)	64.8 (8.7)	78.3 (11.1)	71.4 (5.1)

## 4. Discussion and conclusions

The experimental evaluation shows that the feedback control of the hybrid NMES-exoskeleton system is feasible. Compared to the results presented in Freeman et al. (2012), no learning phase was required to achieve the desired functional movements. Overall, the evaluation shows that it is possible to support the user in performing the drinking task. Because the drinking task was considered the most complex one, we conclude that other tasks are supported with similar effectiveness.

The observed small position errors at the mouth might be corrected by minor head movements to allow the drinking from the cup by means of a straw. When positioning the hand above the object (i.e., the cup handle), in *x^g^*-direction larger errors were observed compared to other directions. But due to the large dimension of the cup handle, the ability to grasp the handle was not restricted. The limited accuracy for placing the hand at objects restricts the possible size and number of objects on the table. Reasons for the observed errors are diverse. One major problem observed is the limited braking torque of 2.5 Nm for the horizontal shoulder rotation that sometimes cannot prevent unwanted slipping. Despite careful placement of the stimulation electrodes, it cannot be avoided that a stimulation of the Deltoid, medial head, generates (besides a desired shoulder extension moment) an unwanted horizontal shoulder rotation moment. If the latter exceeds the torque of the locked horizontal shoulder rotation brake, then slipping occurs for this DoF. With the arm pointing forward, an error in the shoulder horizontal rotation leads to a large hand error in the *x^g^*-direction, especially for the extended arm. In future research, the use of array electrodes for the deltoid muscle might be an option to achieve a more selective stimulation and to avoid such unwanted stimulation effects and slipping. Another solution is to increase the brake torque by re-designing the exoskeleton.

Even when moving to a position given in Cartesian coordinates, the real-time control system is based on angular control. The position errors determined by the exoskeleton angles are purely related to the control system. The errors determined by the Kinect measurements additionally take problems into account that are related to the used kinematic model and coordinate transformations. The current controller design assumes that the exoskeleton/arm-combination represents a rigid body system. This is certainly only an approximation. Moreover, for the calibration of the kinematic model and the coordinate transformation, the arm/hand is moved by an assisting person to twelve arbitrarily chosen different positions in the workspace. Compared to the later use with NMES, no loading/deformation of the exoskeleton by the arm weight takes place. Any deviation from the rigid body assumption causes a position error due to the use of an incorrect forward kinematics. Such an error can only be detected by an external measurement system, like the Kinect, and not by the exoskeleton's internal angle sensors. The larger errors computed from the Kinect measurements compared to the one derived from the exoskeleton sensors are therefore an indicator that the rigid body system assumption is only an approximation.

A shortcoming of the developed system is that elbow extension and shoulder flexion are only induced by gravity. This requires a carefully adjusted weight compensation. Any overcompensation of the weight could drive the arm movement into a dead lock.

Huge advantages of the employed control strategy are its robustness and its simple adaptation to new users/sessions. Only a simple single-input single-output dynamical model needs to be identified for the adaptation of the controller. For all subjects, the same tuning parameters, like rise times and damping factors, have been used for the automatic design of the shoulder extension/flexion controller. In addition to this, the same gains have been applied to the controllers of shoulder horizontal rotation and elbow flexion/extension in all subjects. Due to automated and guided procedures, the system can be set up in a few minutes for the individual user. All individual NMES controllers for the three DoFs include an integrator which allows for the compensation of muscular fatigue as long as the stimulation intensities do not saturate. No deterioration of control performance was observed for the healthy subjects during the five performed trials and from day to day. All these advantages have to be paid by the fact that the movements do not look very physiological and movement sequences are not time optimal (cf. Table 4). However, we hypothesize that this fact is of minor importance for final users, and that the guaranteed functionality overbalances the timing issue for this assistive technology. The personal experience of performing all movements by means of the own muscles is the major advantage compared to robotic approaches for assistance of reaching function (e.g., Maheu et al., 2011). Regular use of the proposed arm neuroprosthesis and, consequently, of the patient's musculature will be health promoting. It will increase muscle strength and might also improve cardiovascular fitness.

In summary, a feedback controlled hybrid NMES-exoskeleton which does not require any residual function at the shoulder and arm level was developed. By combining NMES with the passive exoskeleton for partial arm weight support, muscular fatigue can be significantly reduced since the required amount of muscular force is smaller compared to normal movements. The use of electrically lockable joints reduces the onset of muscular fatigue even further because no muscle function is required to hold the desired position.

The presented study was focusing on the achievable control system performance, which was expected to be maximal for healthy individual due to non-atrophied muscles and the absence of spasticity. During the development of the system, a first test involving one incomplete SCI subject (C4/C5) was performed and showed that the system supported the subject in reaching a cup and bring it to the mouth. The results of this test have been previously published (Pedrocchi et al., 2013). Tests of the final feedback controller on a group of SCI subjects will be performed to observe the feasibility of the system in supporting daily life activities. To obtain successful results, an initial conditioning phase in order to assure that NMES is able to induce some muscle force, and a longer familiarization phase with the system, are envisaged.

## Author contributions

Christian Klauer and Thomas Schauer designed and implemented the real-time NMES control system including interfaces to the central controller and to the sensors and brakes of the exoskeleton. They also derived the kinematic model and set-up for the parameter estimation. Werner Reichenfelser, Jakob Karner, and Margit Gföhler designed and built the passive light-weight exoskeleton. Marta Gandolla and Alessandra Pedrocchi developed the eyetracker interface. Emilia Ambrosini, Simona Ferrante, and Christian Klauer carried out the validation study of the control system including data analysis. Marco Hack and Andreas Jedlitschka developed the Kinect interface and object/hand tracking. Sven Zwicker and Alexander Duschau-Wicke realized the central controller, the overall system integration and the inter-module communication. Alessandra Pedrocchi was the manager of the EU project MUNDUS and responsible for the entire system design. All authors contributed in writing and revising the manuscript.

### Conflict of interest statement

The authors declare that the research was conducted in the absence of any commercial or financial relationships that could be construed as a potential conflict of interest.

## Footnotes

^1^http://www.rtai.org

^2^http://www.scilab.org

^3^http://openrtdynamics.sourceforge.net/

^4^http://sciencestim.sf.net

## Supplementary material

A video of the drinking use case realized by the MUNDUS system showing a healthy subject. The arm movements are generated by means of the described feedback control system. In addition, also a NMES hand module is applied to support the grasping of the object.
